# Sirt3 Ameliorates Oxidative Stress and Mitochondrial Dysfunction After Intracerebral Hemorrhage in Diabetic Rats

**DOI:** 10.3389/fnins.2018.00414

**Published:** 2018-06-19

**Authors:** Jingwei Zheng, Ligen Shi, Feng Liang, Weilin Xu, Tao Li, Liansheng Gao, Zeyu Sun, Jun Yu, Jianmin Zhang

**Affiliations:** ^1^Department of Neurosurgery, The Second Affiliated Hospital, Zhejiang University School of Medicine, Hangzhou, China; ^2^Brain Research Institute, Zhejiang University, Hangzhou, China; ^3^Collaborative Innovation Center for Brain Science, Zhejiang University, Hangzhou, China

**Keywords:** ICH, hyperglycemia, sirt3, mitochondrial dysfunction, oxidative stress

## Abstract

**Aim:** Sirtuin3 (sirt3) plays a pivotal role in improving oxidative stress and mitochondrial dysfunction which directly induced neuronal apoptosis after intracerebral hemorrhage (ICH). Reactive oxygen species (ROS) is also a critical activator in triggering NACHT, LRR, and PYD domains-containing protein 3 (NLRP3) inflammasomes activation which can regulate inflammatory responses in brain. Moreover, hyperglycemia can aggravate the ICH-induced damage. Hence, this study was designed to investigate the mechanisms of neuroprotection of sirt3 in hyperglycemic ICH.

**Methods:** ICH model was established by autologous blood injection. Hyperglycemia was induced by intraperitoneal injection with streptozotocin. Honokiol (HKL, a pharmacological agonist of sirt3) was injected intraperitoneally at doses of 2.5, 5, or 10 mg/kg. Sirt3 small interfering RNA transfection was implemented through intracerebroventricular injection. The expression of sirt3 and its downstream signaling molecules were detected using Western blotting or immunofluorescence staining. Morphological changes of mitochondria were detected by electron microscopy. SH-SY5Y cells were incubated with 10 μM oxyhemoglobin for 48 h to establish an *in vitro* ICH model, and then JC-1 staining was used to determine mitochondrial membrane potential (Δψ*m*).

**Results:** Hyperglycemia could suppress sirt3 expression after ICH when compared with non-diabetic rats. Sirt3 protein expression was decreased to the minimum at 24 h in perihematoma tissues. Electron microscope analysis indicated that hyperglycemic ICH induced extensive mitochondrial vacuolization. HKL attenuated ROS accumulation, adenosine triphosphate reduction, and Δψ*m* through Sirt3–superoxide dismutase 2 (SOD2) and Sirt3–NRF1–TFAM pathway. Sirt3 knockdown could exacerbate the neuronal apoptosis and reverse the positive effects of HKL. Sirt3 activation could decrease NLRP3 and interleukin-1β levels through deacetylating SOD2 and scavenging ROS.

**Conclusion:** HKL protects against hyperglycemic ICH-induced neuronal injury via a sirt3-dependent manner.

## Introduction

Intracerebral hemorrhage (ICH) is a serious public health problem with high rates of death and disability, and it accounts for 10–20% of stroke worldwide (Qureshi et al., 2009; van Asch et al., 2010). Perihematoma edema is the main manifestation of secondary brain injury (SBI) after ICH, and it may cause poor prognosis from increased intracranial pressure or hydrocephalus (Inaji et al., 2003; Murthy et al., 2015). Complicated mechanisms are involved in the formation of brain edema. The initial hematoma induces glutamate release and then leads to oxidative stress and mitochondrial dysfunction. Mitochondrial dysfunction may contribute to the deficiency of adenosine triphosphate (ATP) generation and then result in the failure of cellular pumps causing cytotoxic edema and neuronal apoptosis (Kim-Han et al., 2006; Brunswick et al., 2012; Prentice et al., 2015; Duan et al., 2016). Moreover, mitochondrial dysfunction caused the dysregulation of reactive oxygen species (ROS) homeostasis, ROS accumulation further caused damage in mitochondria, and persistence of vicious circles (Brunswick et al., 2012). NACHT, LRR, and PYD domains-containing protein 3 (NLRP3) inflammasomes are associated with inflammatory responses and cellular injury after ischemic stroke or ICH (Ma et al., 2014). Sufficient evidence demonstrated that ROS is a crucial activator in triggering NLRP3 activation (Chen et al., 2017; Gao et al., 2017). ROS scavenging may be an effective method in inhibiting neuroinflammation. Hence, oxidative stress and mitochondrial dysfunction may be the potential breakthrough in treatment of ICH injury.

Notably, factors such as diabetes mellitus, high blood pressure, and alcohol intake are thought to be predictors of poor outcomes after ICH (Kimura et al., 2007). Several studies also demonstrate that hyperglycemia is associated with severe brain edema and increased level of cell apoptosis in animal models of ICH (Song et al., 2003; Chiu et al., 2013). In addition, hyperglycemia can independently increase the risk of early death in patients with acute spontaneous ICH (Kimura et al., 2007). High blood glucose (HG) can also exacerbate stroke and reperfusion injury through oxidative stress and energy metabolism pathway (Robbins and Swanson, 2014). *In vitro*, hyperglycemia may also results in the activation of ROS and cause the dysregulation of mitochondrial membrane potential which precedes the neuronal apoptosis (Russell et al., 2002). Thus, it remains a great need to find a novel therapeutic target that may help dealing with SBI and hyperglycemia-induced damage in diabetic ICH patients.

Sirtuin3 (sirt3) is a NAD^+^-dependent deacetylase predominately located in mitochondria. Simultaneously, sirt3 could maintain ROS homeostasis through the regulation of a variety of mitochondrial enzymes such as superoxide dismutase 2 (SOD2), which may transform harmful superoxide radicals into nontoxic oxygen or hydrogen peroxide (Qiu et al., 2010; Bause and Haigis, 2013). In addition, sirt3 was also involved in the mitochondrial basal ATP production through the respiratory chain and ATP synthase pathway (Ahn et al., 2008; Giralt and Villarroya, 2012). Moreover, it could react with peroxisome-proliferator-activated receptor-γ co-activator-1α (PGC-1α) which is the crucial controller of mitochondrial biogenesis, and then participated in the regulation of oxidative metabolism (Kong et al., 2010). Notably, a recent study demonstrated that hyperglycemia could inhibit sirt3 expression in retinal capillary endothelial cells and caused worse oxidative stress injury (Gao et al., 2016). A pervious study also indicated that cerebral AQP-4 expression was downregulated after hyperglycemic ICH in rats (Chiu et al., 2013). Gao et al. (2016) thought that hyperglycemia could result in the activation of poly ADP-ribose polymerase (PARP) which competitively utilized the same cofactor (NAD^+^) with sirt3. However, the reason why hyperglycemia can suppress protein expression is still inconclusive. The above-mentioned observations inspired us that sirt3 may play a pivotal role in ICH-induced cerebral injury.

Honokiol (HKL) is a small molecular weight natural compound which can be extracted from *Magnolia grandiflora*. HKL is initially known for its anti-inflammatory, anti-cancerous, and antithrombotic properties (Woodbury et al., 2013). A recent study indicated that intraperitoneal administration of HKL could ameliorate cardiac hypertrophy by activating sirt3. Pharmaceutically, HKL could directly bind to sirt3 and increase sirt3 levels and its enzymatic activity (Pillai et al., 2015). Whether HKL administration can upregulate sirt3 levels and exert neuroprotection in hyperglycemic ICH rats has never been studied. Therefore, this study was designed to: (1) investigate whether hyperglycemia affects sirt3 expression after ICH and (2) explore whether HKL ameliorates oxidative stress and mitochondrial dysfunction via a sirt3-dependent manner.

## Materials and Methods

### Animals

Adult male Sprague-Dawley rats (300–350 g) which were obtained from Slac Laboratory Co., Ltd. (Shanghai, China) were used for this study. A total of 288 rats were used for this study, and the details of grouping information were shown in **Supplementary Figure S1**. Sprague-Dawley rats were raised in triples in plastic cages with controlled temperature and humidity and a 12-h light/dark cycle. All animal experimental protocols were in compliance with the *Guide for the Care and Use of Laboratory Animals* of the National Institutes of Health and were approved by the Institutional Animal Care and Use Committee of Zhejiang University.

### Hyperglycemia and ICH Model

Diabetic model was induced by intraperitoneal injection with streptozotocin (STZ; Sigma-S0130, Sigma–Aldrich Trading Co., Ltd., Shanghai, China) at 60 mg/kg for 3 days before operation (Chiu et al., 2013). Blood glucose was measured by OneTouch Select Test Strips (Johnson & Johnson) with the tail venous blood after overnight fasting, and hyperglycemia was defined as blood glucose >250mg/dl (Liu et al., 2011).

Sprague-Dawley rats were anesthetized with intraperitoneal pentobarbital (50 mg/kg) and experimental ICH surgery was induced in a stereotaxic frame (Stoelting Stereotaxic Instrument). A 1-mm-diameter burr hole was made in the skull (0.2 mm posterior to bregma and 3.5 mm right lateral to midline), then 100 μl fresh autologous blood (catheterization to femoral artery with PE10 tube) was injected into the right basal ganglia (5.5 mm depth below the skull) with a micro-injector within 5 min. In case of blood leakage, the needle would stay for another 10 min after complete injection, then the burr hole was blocked with bone wax (Yang et al., 1994; Song et al., 2003; Chiu et al., 2013). The rats in sham group received all the above-mentioned procedures but the 100 μl saline was injected instead of fresh autologous blood.

### Small Interfering RNA and Intracerebroventricular Injection

*In vivo*: The sirt3 siRNA or scramble siRNA (Thermo Fisher Scientific) mixed with the transfection reagent (Engreen Biosystem Co., Ltd.; 500 pmol/10 μl) was injected into the right lateral ventricle at a rate of 2 μl/min 24 h before ICH (Zheng et al., 2015). The intracerebroventricular injection was performed as previously reported (Xu et al., 2017). After the rats were anesthetized with intraperitoneal pentobarbital (50 mg/kg), a 1-mm-diameter burr hole was made in the skull (1 mm posterior to bregma and 1.5 mm right lateral to midline). Then, 10 μl of the mixture of siRNA and transfection reagent was infused into the right lateral ventricle (3.5 mm depth below the skull). The needle stayed in the brain for another 10 min after injection and the burr hole was blocked with bone wax.

*In vitro*: SH-SY5Y cells were transfected with sirt3 siRNA or control siRNA (Thermo Fisher Scientific) mixed with the transfection reagent (Engreen Biosystem Co., Ltd.; final concentration: 100 nM).

### Drug Administration

*In vivo*: HKL (Purity ≥ 98%, Sigma-H4914, Sigma–Aldrich Trading Co., Ltd., Shanghai, China) was dissolved in dimethyl sulfoxide (DMSO):phosphate-buffered saline (PBS) (1:1) and injected intraperitoneally at a dose of 2.5, 5, or 10 mg/kg immediately at 15 min before inducing ICH and 60 min after ICH (Harada et al., 2012; Sulakhiya et al., 2014). Vehicle rats (ICH + vehicle) received intraperitoneal injections of the same volumes of DMSO:PBS (1:1).

*In vitro*: SH-SY5Y cells were pre-incubated with 10 μM HKL (dissolved in DMSO:PBS = 1:1) for 6 h and then co-treatment with 10 μM oxyhemoglobin (OxyHb, Solarbio, H8020) for an additional 48 h. The control cells received the same volumes of DMSO:PBS (1:1).

### Cell Culture

The human neuroblastoma SH-SY5Y cells were cultured (37°C, 5% CO_2_) in DMEM/F12 (1:1) medium with 15% fetal bovine serum (FBS) and 100 U/ml penicillin. These cells were seeded in 5 ^∗^ 5 cm^2^ cell culture flask at a density range of 4 × 10^6^/well - 5 × 10^6^/well. Treatment of 10 μM OxyHb and 20 mM glucose for 48 h was used to induce an *in vitro* hyperglycemic ICH model (Meng et al., 2018).

### Cell Viability: MTT Assay

Measurement of cell viability was determined using 3-(4,5-dimethylthiazol-2-yl)-2,5-diphenyltetrazolium bromide (MTT) assay kit (Beyotime, Shanghai, China). In brief, the SH-SY5Y cells grown in 96-well plates at a density range of 1 × 10^4^/well - 3 × 10^4^/well with a volume of culture medium measuring 100 μl/well were treated with 10 μl of MTT solution (5 mg MTT in 1 ml 0.01 M PBS, pH = 7.40); the mixture yield was then incubated at 37°C for 2 h. After incubation and discarding the medium, 100 μl of DMSO was added in each well to dissolve insoluble formazan. The cell viability was determined by measuring the absorbance at 570 nm using a microplate reader.

### Measurement of Mitochondrial Membrane Potential (Δψ*m*)

The SH-SY5Y cells planted in 96-well plates at a density range of 1 × 10^4^/well - 3 × 10^4^/well, then these cells were used for measuring Δψ*m*. The Δψ*m* was measured by a JC-1 kit (Beyotime, Shanghai, China) following the manufacturer’s instructions. SH-SY5Y cells were rinsed with PBS and incubated with JC-1 staining solution at 37°C for 20 min. Then, the inverted fluorescence microscope (Olympus, Tokyo, Japan) was used to capture the pictures and calculate the ratio of the red:green fluorescence.

### Measurement of Brain Water Content

As described previously (Xu et al., 2017) to measure the brain water content after ICH, rats were sacrificed under deep pentobarbital anesthesia at 24 h after ICH. We swiftly removed the skull and take out brain tissues with removal of cerebellum and brain stem. Then the right hemispheres were immediately weighed (wet weight), and then dried at 100°C for 24 h in the Electro-Thermostatic Blast Oven. The dry weight of these brain tissues were acquired by reweighting. Ultimately, brain water content was calculated using the following formula: [(wet weight - dry weight)/wet weight] × 100.

### Evaluation of Neurological Deficit

As described previously (Zhao et al., 2006) a combination score consisting of a battery of behavioral tests (foot fault, forelimb placing, postural reflex, and circling tests) was conducted as the comprehensive evaluation of the neurological functional deficits (NDSs). The NDS was performed before and at 1, 3, and 5 days after ICH. The final evaluation of neurological deficit (0–16) was the sum of the scores from the above-mentioned four tests. All the above-mentioned assessment was performed by an independent researcher who was blind to the experimental groups.

### Immunofluorescence Staining and H&E Staining

Rats were sacrificed under deep pentobarbital anesthesia at 24 h after ICH, and then intracardially perfused with 0.1 mmol PBS and 4% paraformaldehyde (PFA). Coronal cryosections were preprocessed with 10% donkey serum and 0.3% triton X-100. Then the brain cryosections were incubated at 4°C with anti-sirt3 antibody (1:200, Abcam, ab86671), cleaved caspase-3 antibody (1:250, Cell Signaling Technology, CST#9661), Iba-1 (1:500, Abcam ab5076), NeuN (1:500, Abcam, ab177487). Twelve hours later, cryosections were incubated with secondary antibody [Thermo Fisher Scientific, Donkey anti-Rabbit IgG (H+L) Alexa Fluor 488, Donkey anti-Mouse IgG (H+L) Alexa Fluor 594, Goat anti-Rabbit IgG (H+L) Alexa Fluor 488]. Then, the fluorescence microscope (Olympus, Tokyo, Japan) was used to capture the images.

As described previously (Xu et al., 2006) brain samples were dissected and embedded in paraffin. Five to eight micrometers of coronal sections were stained with 0.1% cresyl violet hematoxylin and eosin (H&E) and prepared for the subsequent microscope.

### Terminal Deoxynucleotide Transferase dUTP Nick-End Labeling (TUNEL) Stain

A TUNEL staining kit (Roche, Switzerland) was used to assess neuronal apoptosis in basal ganglia (perihematoma) after ICH as previously described (Xu et al., 2017). Apoptosis was defined as TUNEL-positive cells, and counted by an independent individual. The number of TUNEL-positive cells was counted at 200× magnifications in each section.

### Electron Microscopy

Rats were sacrificed under deep pentobarbital anesthesia at 24 h after ICH, and then perfused with 0.9% saline and 4% PFA. 1 mm^3^ fragments of peri-hematoma tissues were obtained from right basal ganglia and then processed with glutaraldehyde (2.5%) at 4°C overnight. As described previously (Fan et al., 2017) the tissues were further handled through a succession of chemical treatment steps (1% osmium tetroxide, distilled water, etc.). Finally, the samples were imbedded in a mixture of propylene oxide and resin (1:1) overnight. After that, the samples were sliced into 100 nm sections and then stained with 4% uranyl acetate and 0.5% lead citrate. The ultrastructure of the basal ganglia was scanned using a transmission electron microscopy (Philips Tecnai 10).

### Measurement of ATP Levels

The ATP level was examined by the luciferase-based ATP assay kit (Beyotime, Shanghai, China). As described in the instruction, the brain tissues were lysed in lysis buffer with centrifugation at 4°C and 12,000 ×*g* for 5 min. Then, the supernatant was extracted for the ATP assay. Before the assay, the ATP working reagents [100 μl; ATP detection reagent:ATP detection reagent diluent (1:9)] were added into a micro-well for 5 min at 37°C. The samples (20 μl) were added and then (2 s later at least) measured by Varioskan Flash (Thermo Fisher Scientific). The ATP concentrations were calculated through the standard curve method. Then the protein levels of different samples were acquired using a detergent-compatible protein assay kit (Bio-Rad, Hercules, CA, United States). Ultimately, the ATP levels were displayed in the form of nanomoles per milligrams.

### Measurement of ROS Level

Levels of ROS in brain tissues were examined using a ROS assay kit (JianCheng, China) according to the manufacturer’s instructions. In brief, samples were lysed in 0.01 mol/l PBS with centrifugation at 4°C, 500 ×*g* for 10 min. Then, the supernatant was extracted for the ROS assay. The supernatant (190 μl) and DCFH-DA (10 μl, 1 mol/l) were mixed in a micro-well at room temperature for 30 min. Afterward, the mixtures were measured by fluorophotometry. Then the protein levels of different samples were acquired using a detergent-compatible protein assay kit (Bio-Rad, Hercules, CA, United States). Ultimately, the ROS levels were displayed in the form of fluorescence/mg protein.

### Western Blot

Six rats in each group at different time points had brain tissues harvested for western blot analysis. Western blot was performed as previously described (Xu et al., 2017). Briefly, frozen perihematoma tissues (basal ganglia) were homogenized in RIPA lysis buffer (Beyotime, Shanghai, China). Then the protein samples were separated by 10% or 12% SDS-PAGE, and transferred onto polyvinylidene fluoride (PVDF) membranes (Millipore). Then, the PVDF membranes were blocked with 5% bovine serum albumin for 1 h and incubated with the primary antibodies overnight, including: anti-sirt3 antibody (1:500, Abcam, ab86671), anti-NRF1 antibody (1:2000, Abcam, ab175932), anti-TFAM (1:1000, Abcam, ab131607), anti-SOD2 (1:5000, Abcam, ab13533), anti-Ac-SOD2 (1:1000, Abcam, ab137037), anti-cleaved caspase-3 (1:1000, CST, cst#9661), anti-Bax (1:1000, CST, cst#2772), anti-Bcl-2 (1:800, SantaCruz, sc-492), anti-NLRP3 (1:1000, ab210491, Abcam), anti-interleukin (IL)-1β (1:2000, Santa Cruz, sc-23459), and β-actin (1:5000, Abcam, ab8226). Then, the PVDF membranes were disposed with relevant secondary antibodies (1:5000) for 1 h at normal temperature. The signals of protein bands were detected with ChemiDoc detection system and quantified using Quantity One software (Bio-Rad, Hercules, CA, United States).

### Statistical Analysis

All data are shown as means ± SD. Data from different groups were compared using one-way or two-way analysis of variance. The Kruskal–Wallis test was used to compare the difference of data in abnormal distribution. Then, Dunn–Bonferroni test was performed for *post hoc* comparison. Statistical Package for the Social Sciences (SPSS; version 22.0) and Prism (version 6.0) software were used for statistical analyses. The *P*-value <0.05 indicated statistical significance.

## Results

### Physiological Data of Diabetic Rats

**FIGURE 1:**
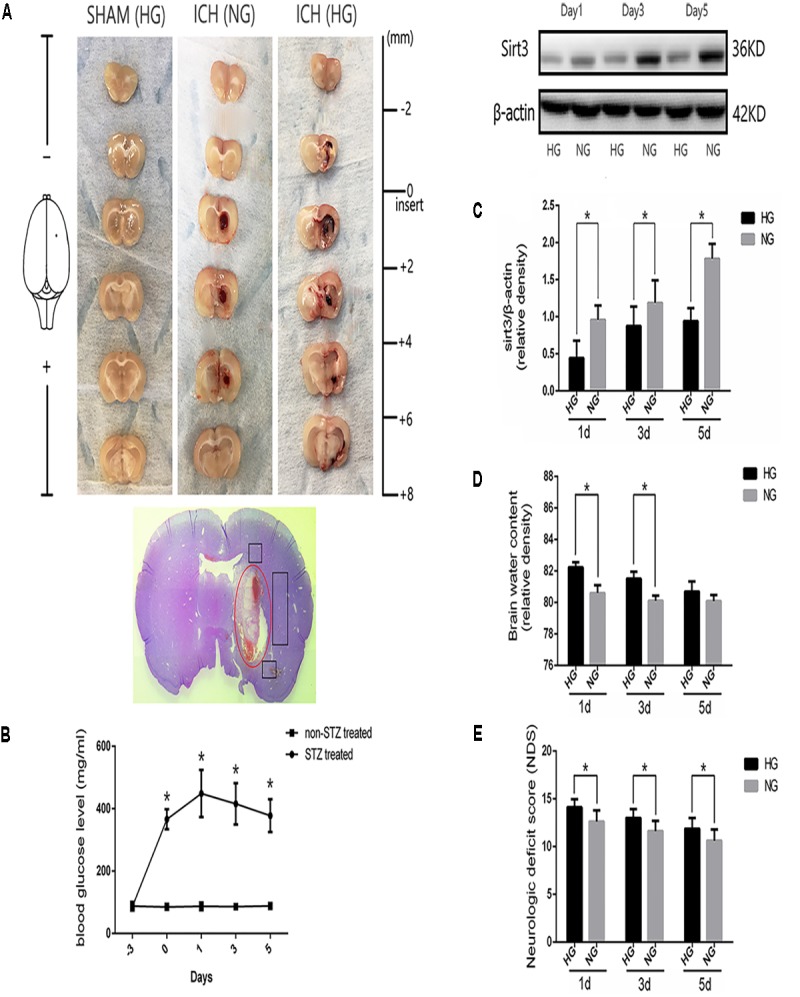
Hyperglycemia suppressed sirt3 expression and aggravated cerebral injury after ICH. **(A)** Representative images of brain sections in each group. HE staining of ICH and the bleeding site (red circle). **(B)** Glucose levels of streptozotocin (STZ) treated and non-STZ-treated rats before and after experimental intracerebral hemorrhage (ICH), ^∗^*P* < 0.05 versus non-STZ-treated group (*n* = 6 in each group). **(C)** Western blotting showed the sirt3 protein expression in HG and NG group on days 1, 3, and 5 after ICH, ^∗^*P* < 0.05 (*n* = 6 in each group). HG, high blood glucose; NG, normal blood glucose. **(D)** The comparison of brain water content in HG and NG group on days 1, 3, and 5 after ICH, ^∗^*P* < 0.05 (*n* = 6 in each group). **(E)** The comparison of neurologic deficit scores in HG and NG group on days 1, 3, and 5 after ICH, ^∗^*P* < 0.05 (*n* = 9 in each group).

Diabetic model was induced by intraperitoneal injection with STZ at 60 mg/kg for 3 days before experimental ICH operation. Blood glucose was measured by glucometer (details were shown in the section “Materials and Methods”) with the tail venous blood. Experimental ICH surgeries were performed in diabetic rats with stereotaxic frame and micro-injector. The representative brain sections from ICH model rats were shown in **Figure 1A**. The results of serial sections indicated that the sagittal length of hematoma in these groups were 0, 5.22 ± 0.62, and 5.52 ± 0.8 mm, respectively (*P* < 0.05, **Supplementary Figure S2**). Since the average sagittal length of rats was 6 mm (Paxinos and Watson, 1998) we calculated the percentages of damage (total lesion length and caudate nucleus distribution), and the results also showed no significant difference between ICH [normal blood glucose (NG)] and ICH [high blood glucose (HG)] group (86.9 ± 0.1% versus 0.92 ± 0.1%, *P* < 0.05). These results indicate that the ICH model is stability and reproducibility. As shown in the H&E staining picture, the perihematoma regions (black box) were used for examining the immunofluorescent staining. Significant differences were exhibited between STZ-treated and non-STZ-treated rats. The average blood glucose levels were 402.1 ± 64.2 and 86.7 ± 8.8 mg/dl, respectively, and this increase was maintained throughout 5 days in our research (**Figure 1B**). Other physiological parameters showed no significant differences between each group (**Supplementary Figure S3**).

### Hyperglycemia Suppressed Sirt3 Expression and Aggravated Brain Injury After ICH

To investigate whether sirt3 expression is influenced by hyperglycemia. Western blotting was performed to explore the differences of sirt3 expression between non-diabetic and diabetic rats after ICH. The results showed that sirt3 expression was suppressed on days 1, 3, and 5 after ICH in HG group when compared with normal blood glucose (NG) group (*P* < 0.05, **Figure 1C**).

Intracerebral hemorrhage-induced brain injury was measured by neurological deficit scores and brain water content. Brain water content of lesioned hemisphere was 82.2 ± 0.32% in HG group and 80.4 ± 0.65% in NG group on day 1 after ICH (*P* < 0.01, **Figure 1D**). On day 3, the result was 81.5 ± 0.44% in HG group and 80.1 ± 0.33% in NG group (*P* < 0.01). However, no significant difference was detected on day 5 after ICH between HG group: 80.1 ± 0.4% and NG group: 79.9 ± 0.4% (*P* > 0.05).

In addition, neurological deficit scores (**Figure 1E**) showed: 14.1 ± 0.8 in HG group and 12.6 ± 1.2 in NG group on day 1 after ICH (*P* < 0.05); 13.0 ± 0.9 in HG group and 11.6 ± 1.1 in NG group on day 3 (*P* < 0.05); 11.9 ± 1.1 in HG group and 10.6 ± 1.2 in NG group on day 5 (*P* < 0.05).

The above-mentioned findings indicated that hyperglycemia suppressed sirt3 expression after ICH may be one of the reasons why hyperglycemia could deteriorate ICH-induced cerebral injury.

### Sirt3 Expression in Diabetic Rats After ICH

**FIGURE 2:**
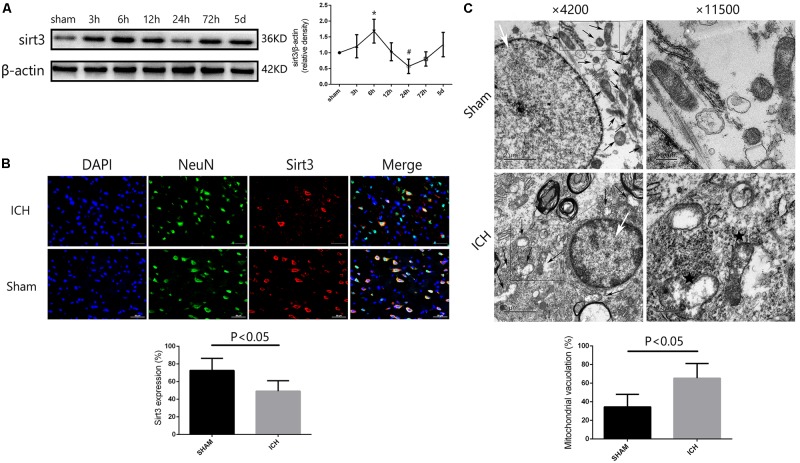
Sirt3 protein expression and morphological changes of mitochondria after hyperglycemic ICH. **(A)** Western blotting showed that sirt3 protein expression was significantly decreased to the minimum at 24 h after ICH. Before that, sirt3 expression was reactively upregulated at 6 h after ICH, ^∗^*P* < 0.05 versus sham group, **^#^***P* < 0.05 versus the rest groups (*n* = 6 in each group at different time points). **(B)** Immunofluorescence staining showed the location of sirt3 protein in basal ganglia after hyperglycemic ICH at 24 h. Sirt3 expression was downregulated after ICH when compared with sham group (*P* < 0.05, 18 cryosections from 6 rats were calculated in each group, scale bars: 50 μm). **(C)** Transmission electron microscopy images of neuronal apoptosis and mitochondrial ultrastructure (white arrow: cell nucleus; black arrow: mitochondria). The ratio of mitochondrial vacuolization was increased after ICH when compared with sham group (*P* < 0.05, 18 TEM images from 6 rats were calculated in each group).

To investigate the time course of sirt3 expression in hyperglycemic ICH. We analyzed the sirt3 protein expression at 0 (sham group), 3, 6, 12, 24, 72, and 120 h after ICH. As shown in **Figure 2A**, the results of time course indicated that the protein expression of sirt3 was decreased to the minimum at 24 h after ICH (*P* < 0.05 versus the rest groups). Before that, sirt3 expression was increased at 6 h after ICH (*P* < 0.05 versus sham group).

Double immunofluorescence staining was performed to assess location of sirt3 expression. We found that sirt3 expression was decreased after ICH when compared with sham group (**Figure 2B**, *P* < 0.05).

### Electron Microscopy Analysis

In the results of transmission electron microscopy, obvious changes were found in hyperglycemic ICH rats when compared with non-ICH diabetic rats. The magnifications were 4200 or 11,500 times. As shown in **Figure 2C**, (1) the asterisked mitochondria (the left one with damaged mitochondrial crest) showed serious vacuolization and swelling, the right one was a normal mitochondria with integrated mitochondrial crest; (2) the nuclear chromatin concentration and margination were also seen after hyperglycemic ICH; and (3) the ratio of mitochondria vacuolization (vacuolated**/**total mitochondria nearby the cell nuclear) was also calculated, the results indicated that hyperglycemic ICH induced extensive mitochondrial vacuolization and mitochondria swelling (*P* < 0.05).

### HKL Increased the Expression of Sirt3 and Its Downstream Signaling Molecules After Hyperglycemic ICH

**FIGURE 3:**
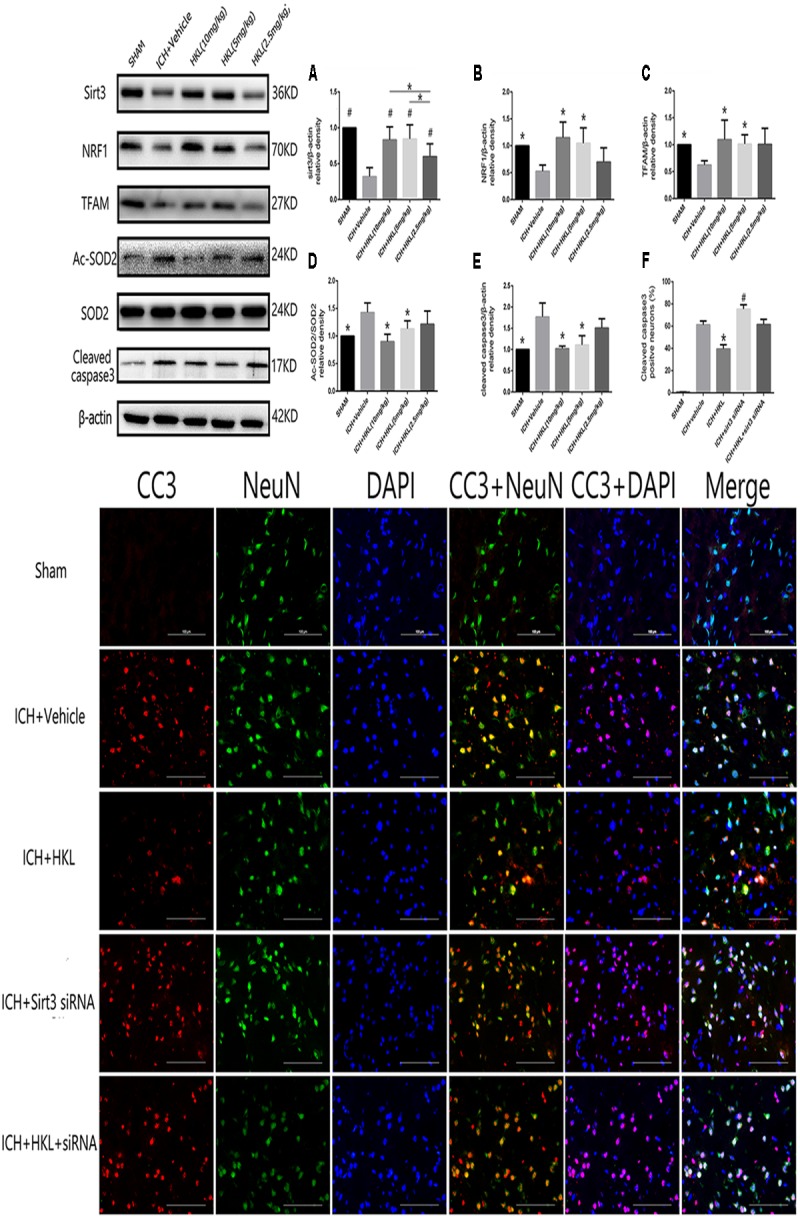
HKL mediated the expression of sirt3 and its downstream signaling molecules after hyperglycemic ICH. **(A)** Sirt3 protein expression was significantly enhanced by HKL in a dose-dependent manner. **^#^***P* < 0.05 versus ICH + vehicle group, ^∗^*P* < 0.05 (*n* = 6 in each group). **(B)** NRF1 expression, ^∗^*P* < 0.05 versus ICH + vehicle group (*n* = 6 in each group). **(C)** TFAM expression, ^∗^*P* < 0.05 versus ICH + vehicle group (*n* = 6 in each group). **(D)** *Ac-SOD2/SOD2 ratio* (*Ac-SOD2*, acetylated SOD2), ^∗^*P* < 0.05 versus ICH + vehicle group (*n* = 6 in each group). **(E)** Cleaved caspase3 expression, ^∗^*P* < 0.05 versus ICH + vehicle group (*n* = 6 in each group). **(F)** Representative co-labeling cleaved-caspase3/NeuN images of ipsilateral basal ganglia. Quantification of cleaved caspase3-positive neurons (% = cleaved caspase3-positive neurons/total neurons), *^∗^P* and ^#^*P* < 0.05 versus the rest groups (*n* = 6 in each group). Scale bars: 100 μm.

Three different doses of HKL were selected to identify the effective concentration for ICH. As shown in **Figure 3A**, Sirt3 levels were significantly enhanced by HKL in a dose-dependent manner (*P* < 0.05 versus ICH + vehicle group). Meanwhile, sirt3 levels were higher in high and medium dosage group (10, 5 mg/kg) than that in low dosage group (2.5 mg/kg).

Mitochondria played an important role in energy production and cell death, nuclear respiratory factor 1–mitochondrial transcription factor A (NRF1–TFAM) was a crucial signal pathway in mitochondrial biogenesis (Li et al., 2017). The present study indicated that NRF1 expression was significantly increased in medium and high dosage group (*P* < 0.05 versus ICH + vehicle group, **Figure 3B**), and its downstream protein-TFAM was also upregulated after HKL treatment (*P* < 0.05 versus ICH + vehicle group, **Figure 3C**). Sirt3 could directly bind and deacetylate SOD2, which plays a crucial role in scavenging ROS (Kawamura et al., 2010; Qiu et al., 2010; Tao et al., 2010). Consist with previous studies (Pillai et al., 2015; Yu et al., 2017; Zhai et al., 2017), the present study indicated that HKL could decrease ac-SOD2/SOD2 ratio (*P* < 0.05 versus ICH + vehicle group, **Figure 3D**).

As shown in **Figure 3E**, the results indicated that HKL (10 or 5 mg/kg) could protect against neuronal apoptosis via decreasing cleaved caspase3 expression (*P* < 0.05 versus ICH + vehicle group). Hence, we considered 10 mg/kg HKL as the optimal drug concentration.

### Sirt3 Knockdown Reversed the Positive Effects of HKL in the Expression of Sirt3 and Its Downstream Molecules After Hyperglycemic ICH

Sirt3 siRNA was used to further investigate the effects of sirt3 in diabetic rats after hyperglycemic ICH. The double immunofluorescence staining results indicated that cleaved caspase3 levels were significantly decreased after HKL treatment (*P* < 0.05 versus ICH + vehicle group, **Figure 3F**). Sirt3 siRNA transfection could aggravate the upregulation of cleaved caspase3 and block the positive effects of HKL (*P* < 0.05 versus ICH + HKL group).

**FIGURE 4:**
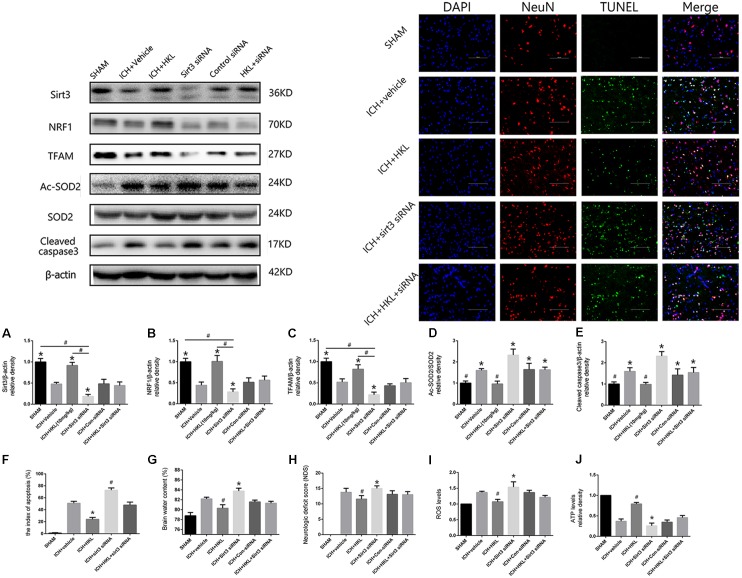
Sirt3 knockdown abolished the positive effects of HKL and exacerbated cerebral injury. **(A)** Sirt3 expression. **(B)** NRF1 expression. **(C)** TFAM expression. *^∗^P* < 0.05 versus the rest three groups, *^#^P* < 0.05 (*n* = 6 in each group). **(D)** Ac-SOD2/SOD2 ratio. **(E)** Cleaved caspase3 expression. *^∗^P* < 0.05 versus the rest two groups, ***^#^****P* < 0.05 versus the rest four groups (*n* = 6 in each group). **(F)** Representative co-labeling TUNEL/NeuN pictures of ipsilateral basal ganglia. Quantification of TUNEL-positive neurons (the index of apoptosis = TUNEL-positive neurons/total neurons, *n* = 6 in each group), Scale bars: 100 μm. **(G)** Brain water content. **(H)** Neurologic deficit scores. **(I)** ROS levels. **(J)** ATP levels *^∗^P* and *^#^P* < 0.05 versus the rest groups (*n* = 6 in each group).

As shown in **Figure 4A**, sirt3 protein levels were significantly decreased in ICH + sirt3 siRNA group when compared with other groups (*P* < 0.05). Meanwhile, sirt3 siRNA transfection could block the activating effects of HKL in sirt3 expression (*P* < 0.05 versus ICH + HKL group). NRF1 and TFAM levels were also significantly decreased after sirt3 knockdown (*P* < 0.05 versus ICH + vehicle group, **Figures 4B,C**). HKL also failed to upregulate NRF1 and TFAM expression in ICH + HKL + siRNA group (*P* < 0.05 versus ICH + HKL group). In **Figure 4D**, Ac-SOD2/SOD2 ratio was significantly increased in ICH + sirt3 siRNA group when compared with ICH + HKL group and ICH + vehicle group (*P* < 0.05). Moreover, sirt3 siRNA could block the effects of HKL in Ac-SOD2/SOD2 ratio (*P* < 0.05 versus ICH + HKL group). Furthermore, in **Figure 4E**, the results indicated that cleaved caspase3 was distinctly increased in ICH + sirt3 siRNA group (*P* < 0.05 versus ICH + vehicle group). In ICH + HKL + sirt3 siRNA group, the anti-apoptosis effects of HKL were blunted after sirt3 silenced (*P* < 0.05 versus ICH + HKL group).

### TUNEL Staining

Further TUNEL staining was used to confirm the effects of HKL on neuronal survival (**Figure 4F**). TUNEL-positive cells were significantly increased in ICH + vehicle group compared with sham group (*P* < 0.05). Greatly increase of TUNEL-positive cells were found in ICH + sirt3 siRNA group (*P* < 0.05 versus ICH + vehicle group). Moreover, sirt3 agonist (HKL) could decrease ICH-induced neuronal apoptosis (*P* < 0.05 versus ICH + vehicle group), and such effects could be blocked by sirt3 siRNA (*P* < 0.05 versus ICH + HKL group).

### Sirt3 Knockdown Aggravated Cerebral Injury via Exacerbating Oxidative Stress and Mitochondrial Dysfunction

Experimental ICH induction could distinctly increase brain water content in ipsilateral hemisphere 82.2 ± 0.33% at 24 h when compared with sham group 78.8 ± 0.6% (*P* < 0.05, **Figure 4G**). Brain water content was further elevated after sirt3 siRNA transfection 83.6 ± 0.7% (*P* < 0.01 versus the rest groups). HKL could alleviated brain edema 80.25 ± 0.33% after ICH (*P* < 0.01 versus the rest groups), and such effects were reversed after sirt3 knockdown 81.43 ± 0.48% (*P* < 0.01 versus ICH + HKL group).

In addition, neurological deficits were more severe in ICH + vehicle group (13.8 ± 1.3) than that of sham group (*P* < 0.05, **Figure 4H**), and sirt3 knockdown (15.0 ± 0.8) could aggravate neurological deficits after ICH (*P* < 0.01 versus ICH + vehicle group). HKL could decrease neurological deficit scores (11.56 ± 1.13, *P* < 0.01 versus ICH + vehicle group), and the positive effect of HKL was reversed after sirt3 knockdown (13.0 ± 1.0, *P* < 0.01 versus ICH + HKL group).

As shown in **Figure 4I**, ROS levels were significantly decreased in HKL-treated group (*P* < 0.05 versus ICH + vehicle group) which were consistent with the decrease of Ac-SOD2/SOD2 ratio. In addition, ROS accumulation was more severe in ICH + sirt3 siRNA group (*P* < 0.05 versus ICH + vehicle group) after sirt3 siRNA transfection. The positive effect of HKL in attenuating ROS accumulation was reversed after sirt3 knockdown (*P* < 0.05 versus ICH + HKL group).

Upregulation of sirt3 expression also enhanced ATP levels when compared with ICH group (*P* < 0.05, **Figure 4J**). Because, sirt3 activation upregulated NRF1 and TFAM expression that could promote mitochondrial biogenesis and then facilitate ATP generation. After sirt3 silenced, ATP reduction was more severe in ICH + sirt3 siRNA group than that of ICH group (*P* < 0.05). Meanwhile, in ICH + sirt3 siRNA + HKL group, sirt3 siRNA transfection could reverse the positive impact of sirt3 agonist in preserving against ATP reduction (*P* < 0.05 versus ICH + HKL group).

### Sirt3 Activation Improved Mitochondrial Permeability Potential and Cell Viability in SH-SY5Y Cells Exposed to OxyHb

**FIGURE 5:**
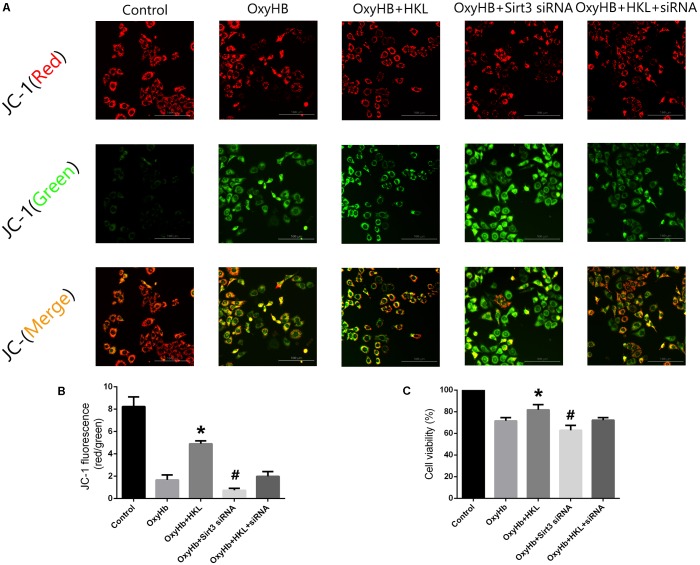
Measurement of Δψ*m* and cell viability in SH-SY5Y cells. **(A)** Representative JC-1 staining pictures of SH-SY5Y cells. **(B)** Quantification of JC-1 fluorescence intensity (red/green florescent area), Scale bars: 100 μm. **(C)** Cell viability. ^∗^*P* and **^#^***P* < 0.05 versus the rest groups (*n* = 6 in each group).

Mitochondrial membrane potential (Δψ*m*) was determined by JC-1 staining. As shown in **Figure 5A**, normal membrane potential indicated red fluorescence intensity in control group. Treatment of 10 μM OxyHb and 20 mM glucose increased green fluorescence intensity which represented the decline of Δψ*m* in SH-SY5Y cells. Pre-incubation with HKL attenuated OxyHb-induced collapse of Δψ*m*, but sirt3 siRNA transfection reversed such effects. The results in scramble siRNA group indicated no significant difference between OxyHb group (data not shown). Quantification of JC-1 fluorescence intensity (red/green florescent area) was shown in **Figure 5B**.

Moreover, the effects of sirt3 activation in protecting SH-SY5Y cells from OxyHb-induced cell injury were evaluated by MTT assay (**Figure 5C**). The viability of SH-SY5Y cells was significantly increased after the treatment of HKL (81.76 ± 4.9%) when compared with OxyHb group (71.59 ± 3.01%). Sirt3 siRNA transfection could decrease the viability (62.9 ± 4.7%) and reverse the positive effect of HKL (72.22 ± 2.3%). The results in scramble siRNA group indicated no significant difference between OxyHb group (data not shown).

### Sirt3 Attenuated Neuroinflammation Through Sirt3/ROS/NLRP3 Pathway

**FIGURE 6:**
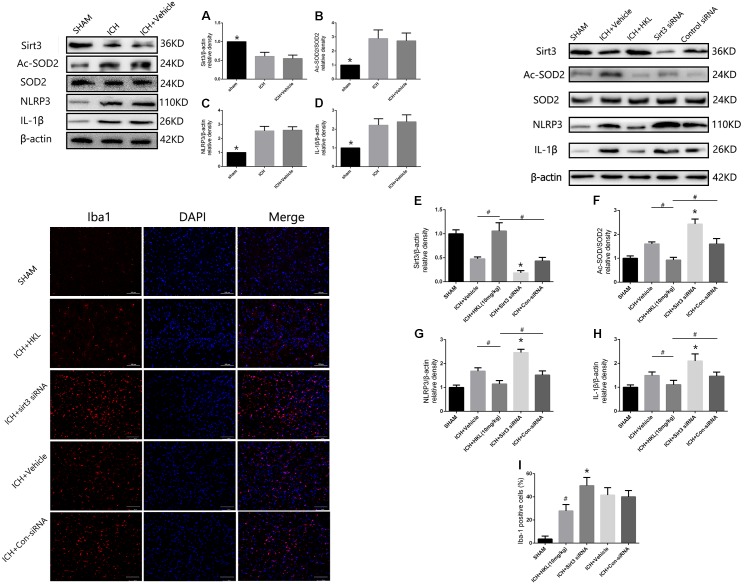
Sirt3 attenuated neuroinflammation through Sirt3–ROS–NLRP3 signaling pathway. **(A)** Sirt3 expression. **(B)** Ac-SOD2/SOD2 ratio. **(C)** NLRP3 expression. **(D)** IL-1β expression. ^∗^*P* < 0.05 versus the rest groups. **(E)** Sirt3 expression. **(F)** Ac-SOD2/SOD2 ratio. **(G)** NLRP3 expression. **(H)** IL-1β expression. ^∗^*P* < 0.05 versus the rest groups, **^#^***P* < 0.05 (*n* = 6 in each group). **(I)** Quantification of Iba-1 positive cells (% = Iba-1-positive cells/total cells, ^∗^*P* < 0.05 versus the rest groups, **^#^***P* < 0.05 versus the rest groups). Scale bars: 100 μm.

The present study indicated that Sirt3 expression was downregulated after hyperglycemic ICH, and then caused the increase of Ac-SOD2/SOD2 ratio which could aggravate ROS accumulation (**Figures 6A,B**). Since the mitochondrial ROS accumulation is one of the critical causes in triggering NLRP3 activation (Ma et al., 2014; Chen et al., 2017). The NLRP3 inflammasome plays a crucial role in neuro-inflammation after ICH, and NLRP3 activation can further lead to the release of pro-inflammatory cytokine IL-1β (Ma et al., 2014). NLRP3 and IL-1β levels were significantly increased after experimental ICH (**Figures 6C,D**). Sirt3 agonist (HKL) could significantly increase the sirt3 protein levels, and then deacetylated SOD2. Thereby, Ac-SOD2/SOD2 ratio and ROS levels were decreased after sirt3 upregulation, and then NLRP3 and IL-1β levels were suffered a corresponding decrease (**Figures 6E–H**). Inversely, sirt3 siRNA transfection could exacerbate neuroinflammation through upregulating sirt3 expression and consequently increasing NLRP3, IL-1β levels, and Ac-SOD2/SOD2 ratio.

In addition, the immunofluorescence staining results indicated that the amount of Iba-1-positive cells was significantly decreased when compared with ICH + Vehicle group (*P* < 0.05, **Figure 6I**). Sirt3 siRNA transfection could reverse such results and extremely increased the quantity of Iba-1-positive cells (*P* < 0.05 versus ICH + vehicle group, **Figure 6I**). It demonstrated that sirt3 attenuated neuroinflammation through sirt3/ROS/NLRP3 pathway.

## Discussion

In present study, we first demonstrated that: (1) hyperglycemia can aggravate the downregulation of sirt3 in animal models of ICH; (2) HKL ameliorates oxidative stress and mitochondrial dysfunction via a sirt3-dependent manner after hyperglycemic ICH; and (3) Sirt3 activation could also decrease NLRP3 and IL-1β levels through deacetylating SOD2 and scavenging ROS.

Perihematomal edema is the main manifestation of SBI, and it is significantly associated with risk of poor functional outcomes (Murthy et al., 2015). The initial hematoma leads to mitochondrial dysfunction through the induction of glutamate release. Mitochondrial deficits cause ATP reduction and then result in the failure of cellular pumps causing cytotoxic edema and cell death (Kim-Han et al., 2006; Brunswick et al., 2012). Mitochondrial dysfunction may also lead to the overproduction of ROS which can induce inflammation and apoptosis (Prentice et al., 2015; Duan et al., 2016). The edema and ROS result in more cell apoptosis, further glutamate release, and persistence of vicious circles. In addition, hyperglycemia may also result in the activation of ROS and cause the dysregulation of mitochondrial membrane potential which precedes the neuronal apoptosis (Russell et al., 2002). Hence, oxidative stress and mitochondrial dysfunction are thought to play an important role in hyperglycemic ICH.

Interestingly, our research data indicated that hyperglycemia could suppress sirt3 expression in HG and NG group on days 1, 3 and 5, and then cause more serious cerebral damage after ICH. This finding was consistent with the previous study that hyperglycemia could inhibit sirt3 expression in retinal capillary endothelial cells (Gao et al., 2016). Moreover, the results of sirt3 expression showed no significant difference between ICH (NG) and ICH (HG) group at other time points. The details of the comparison were shown in **Supplementary Figure S4**. A pervious study also demonstrated that cerebral AQP-4 expression was downregulated in hyperglycemic ICH (Chiu et al., 2013). However, the reason why hyperglycemia can suppress protein expression is still unknown. Gao et al. (2016) thought that hyperglycemia could result in the activation of PARP which competitively utilized the same cofactor (NAD^+^) with sirt3. Hence, hyperglycemia-induced PARP activation was thought to be one of the reasons that caused the downregulation of sirt3 (Gao et al., 2016). Since the downregulation of sirt3, its neuroprotective effects were receded. Therefore, that may be one of the reasons why hyperglycemia can aggravate brain edema and neuronal apoptosis.

Mitochondrial dysfunction includes ATP reduction, damaged mitochondrial biogenesis, disordered membrane potential, and ROS accumulation (Pieczenik and Neustadt, 2007; Bhatti et al., 2017). Sirt3 is involved in the mitochondrial basal ATP production through the ETC and ATP synthase pathway (Ahn et al., 2008; Giralt and Villarroya, 2012). The electron transport chain (ETC) can create the mitochondrial membrane potential (Δψ*m*) which is essential for ATP generation (Bause and Haigis, 2013). As we know, intracellular ATP level is essential for neuronal survival. ICH-induced mitochondrial deficits may contribute to ATP reduction and then result in the failure of cellular pumps causing neuronal apoptosis. The present study indicated that HKL improved Δψ*m* via activating sirt3. Moreover, ATP levels in perihematoma tissues were significantly decreased after hyperglycemic ICH injury. HKL could enhance ATP levels and sirt3 siRNA transfection could reverse such effects. In addition, we also found that NRF1 and TFAM expression were altered along with the alteration of sirt3 expression. Notably, NRF1 and TFAM are proven to play an important role in mitochondrial biogenesis (Li et al., 2017). As the upstream molecule of TFAM, NRF1 activation can increase TFAM expression and exert functions such as mitochondrial DNA transcription, maintenance, replication, and repair (Picca and Lezza, 2015). A recent study also demonstrated that AMPK-PGC-1α-SIRT3 pathway is involved in the regulation of NRF1 and its downstream TFAM (Yu et al., 2017). Given the above-mentioned findings from previous and present studies, sirt3 may protect against neuronal apoptosis via improving ATP generation and mitochondrial biogenesis after hyperglycemic ICH.

Sirt3 has been shown to bind and deacetylate several metabolic and respiratory enzymes that participate in the regulation of ETC function (Giralt and Villarroya, 2012; Bause and Haigis, 2013). ETC plays a pivotal role in mitochondrial ROS production because of numerous ${\text{O}}_{2}^{-}$ generated by complexes I and III. Sirt3 could maintain ROS homeostasis through SOD2, which may transform harmful superoxide radicals into nontoxic oxygen or hydrogen peroxide (Qiu et al., 2010; Bause and Haigis, 2013; Gao et al., 2016). The present study demonstrated that HKL decreased cellular Ac-SOD2/SOD2 ratio in a sirt3-dependent manner after hyperglycemic ICH. The deacetylation by sirt3 is essential for SOD2 activity and ROS elimination. Our research indicated that AcSOD2/SOD2 ratio was increased, and HKL could decrease AcSOD2/SOD2 ratio and increase SOD2 activity. Sirt3 siRNA transfection reversed such effects and aggravated oxidative stress injury after hyperglycemic ICH. In conclusion, sirt3 activation can also attenuate oxidative stress injury through deacetylating SOD2 after hyperglycemic ICH.

Furthermore, several studies have demonstrated that ROS is a critical activator which directly or indirectly induce NLRP3 activation (Zhou et al., 2010; Ma et al., 2014; Chen et al., 2017). A recent study has also shown that NLRP3-induced vascular inflammation can be inhibited through sirt3/SOD2/mtROS pathway (Chen et al., 2017). Notably, NLRP3 is associated with inflammatory responses which play a crucial role in SBI after ICH and ischemic stroke (Ma et al., 2014; Gao et al., 2017). Activation of innate inflammatory responses causes the release of inflammatory cytokines such as IL-1β and tumor necrosis factor-α (TNF-α) (Brunswick et al., 2012; Zhou et al., 2014). These factors further activate the downstream pathway which may cause BBB disruption and vasogenic edema, and ultimately lead to the extensive cell death (Xi et al., 2006; Wang and Dore, 2007). Hence, ROS scavenging may be an effective method in inhibiting NLRP3 activation and attenuating the relevant inflammation. Consistent with the above-mentioned studies, the present study indicated that sirt3 activation could deacetylate SOD2 and then scavenge excess ROS after hyperglycemic ICH. Ultimately, NLRP3 and IL-1β were decreased in a sirt3/SOD2/ROS-dependent pathway. These results implicated that sirt3 could mediate ICH-induced neuroinflammation through sirt3/ROS/NLRP3 signaling pathway.

## Conclusion

In summary, all these findings reveal that sirt3 acts as a crucial factor in improving brain edema and neuronal apoptosis after hyperglycemic ICH. The neuroprotective mechanism of sirt3 mainly depends on the resistance to oxidative stress, mitochondrial dysfunction via SOD2 and NRF1–TFAM pathway. Meanwhile, the Sirt3/ROS/NLRP3 pathway also participates in mediating inflammatory reaction after hyperglycemic ICH. Such findings implicate a novel therapeutic target for hyperglycemic ICH.

## Author Contributions

JMZ is the principal investigator. JWZ and JY contributed to the study design, performance, and manuscript draft. FL, ZS, and LS analyzed the experimental data. WX, TL, and LG revised the manuscript and polished the language.

## Conflict of Interest Statement

The authors declare that the research was conducted in the absence of any commercial or financial relationships that could be construed as a potential conflict of interest.

## Supplementary Material

FIGURE S1 Grouping information.

FIGURE S2 Stereological analysis.

FIGURE S3 Physiological parameters.

FIGURE S4 Sirt3 expression in HG and NG group at different time points after ICH.
