# Supplementary figures and images for: Sirt3 Ameliorates Oxidative Stress and Mitochondrial Dysfunction After Intracerebral Hemorrhage in Diabetic Rats
Source: Front Neurosci. 2018 Jun 19;12:414. doi: 10.3389/fnins.2018.00414 (PMC6018086; doi:10.3389/fnins.2018.00414)

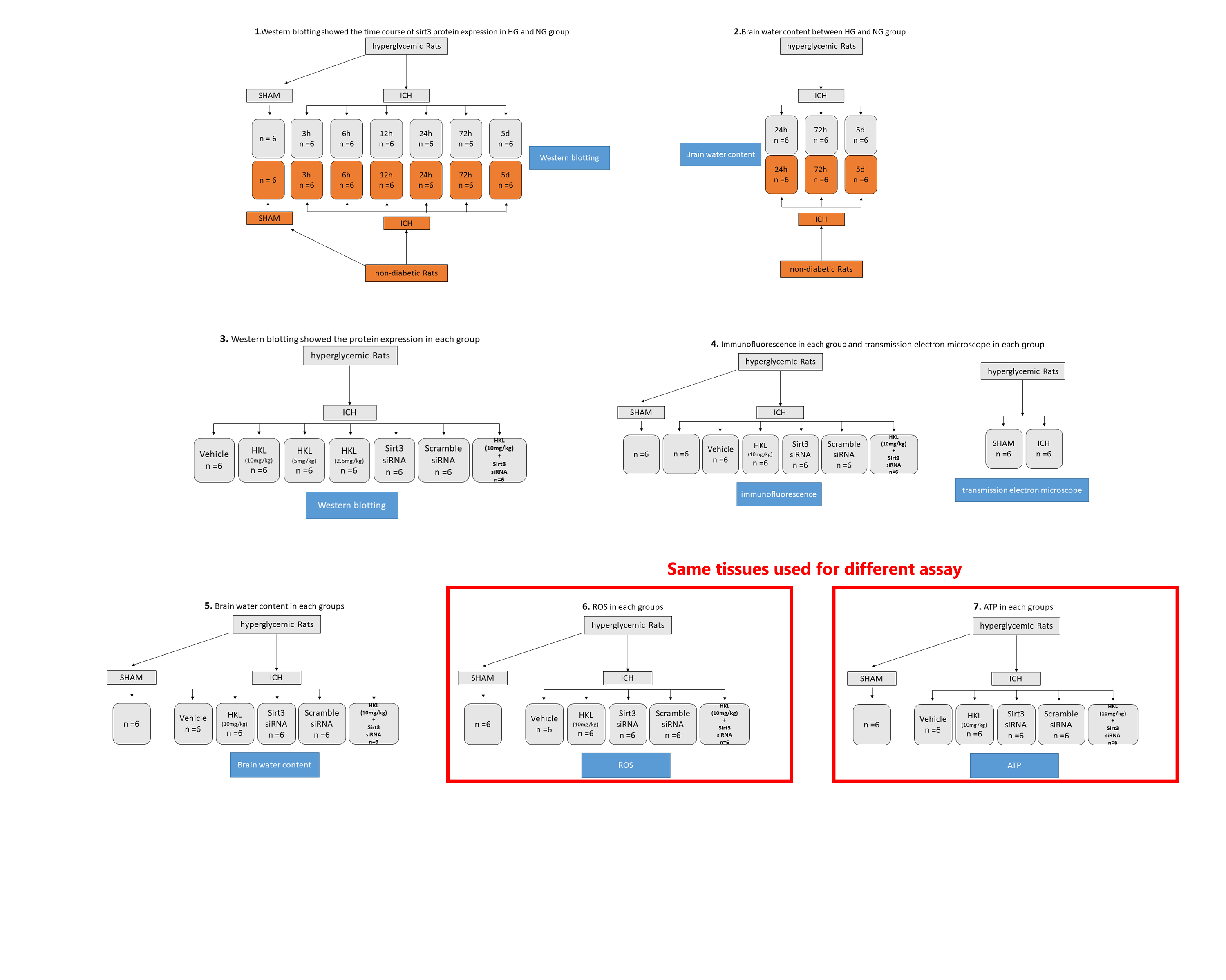

Supplement: FIGURE S1 — Grouping information. [file Image_1.TIF]

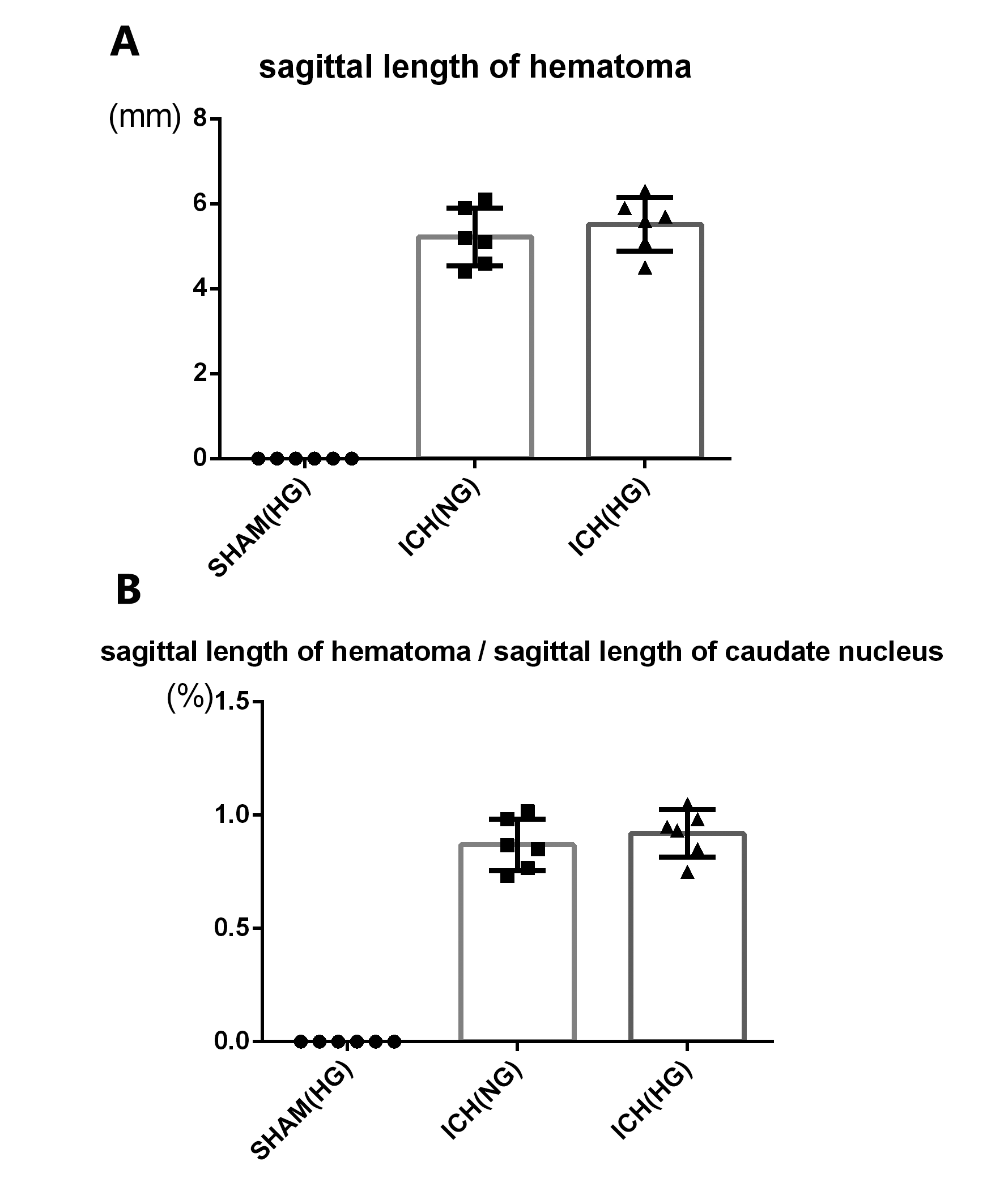

Supplement: FIGURE S2 — Stereological analysis. [file Image_2.TIF]

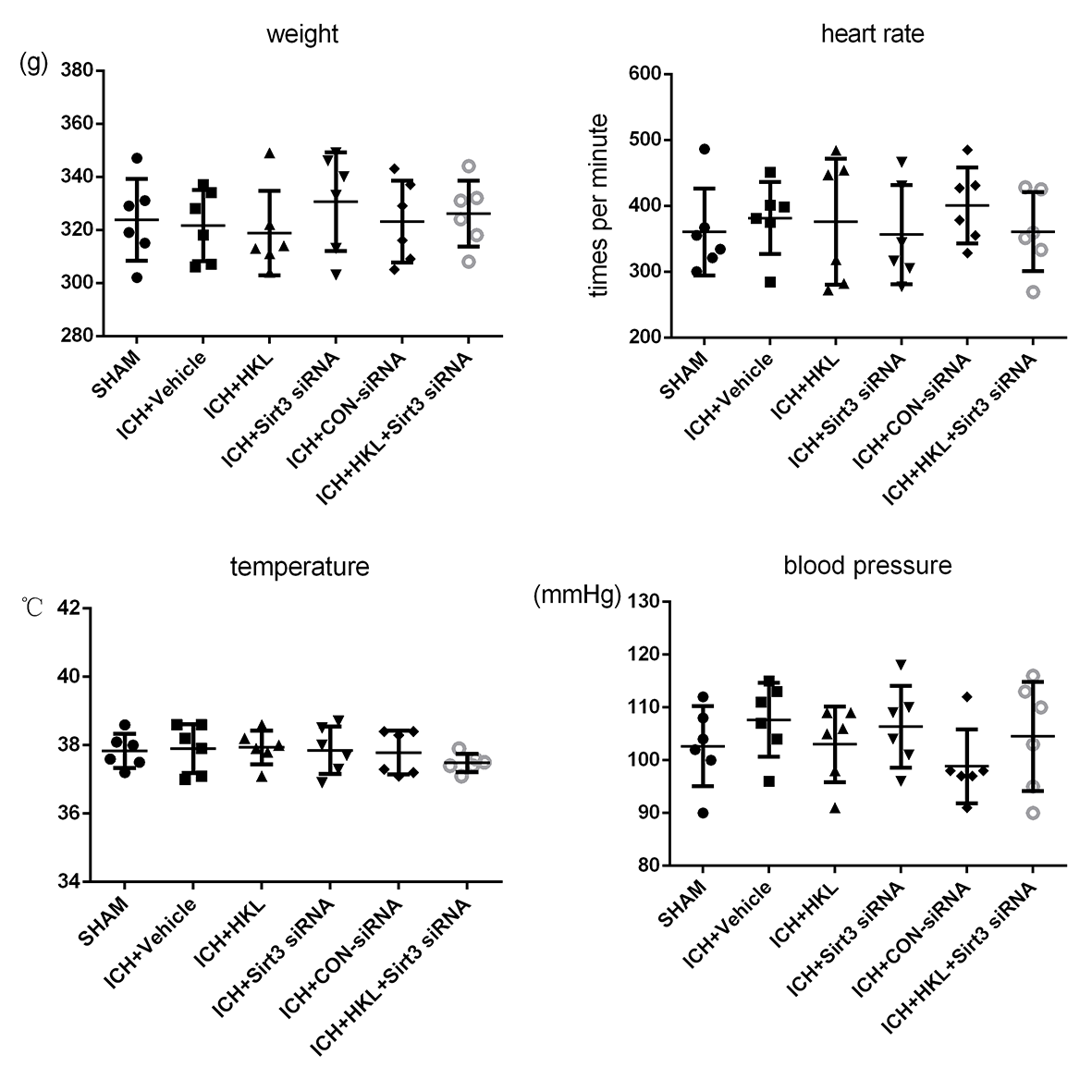

Supplement: FIGURE S3 — Physiological parameters. [file Image_3.TIF]

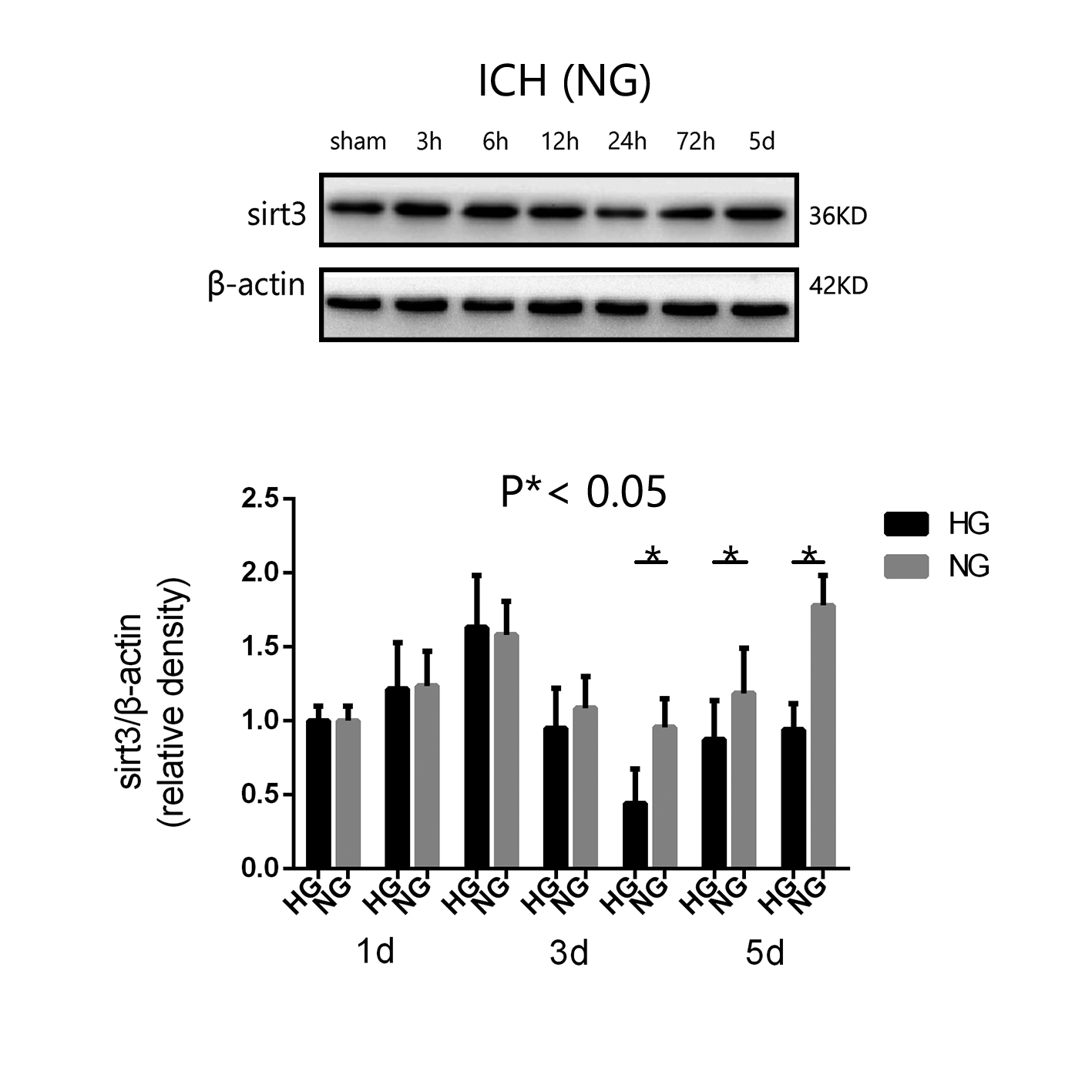

Supplement: FIGURE S4 — Sirt3 expression in HG and NG group at different time points after ICH. [file Image_4.TIF]
